# Medico-Chirurgical Transactions; Published by the Royal Medical and Chirurgical Society of London

**Published:** 1836-07

**Authors:** 


### Art. VII.

*Medico-Chirurgical Transactions; published by the Royal Medical and Chirurgical Society of London. Volume the Nineteenth.—London,* 1835. 8vo. pp. 438.

Since the publication of the last volume of their Transactions, the Society has received the honour of his Majesty's royal charter of incorporation, constituting the Society the Royal Medical and Chirurgical Society of London; designating his Majesty as the patron of the Society, and creating its members Fellows of the chartered body. The Charter and Bye-laws of the Society are given as an introduction to the present volume, with a list of the subscriptions to defray the expenses of the charter. No proof can now be wanted of the very high rank which this Society has always maintained among the scientific bodies of this country; and, if it were required, it would be afforded by the honour of having received the royal patronage, which is never conferred upon any society without deliberate enquiry into the claims which exist for so flattering a distinction. In virtue of their incorporation, the Society is relieved from the regal restrictions of the statutes of Mortmain, and may now "receive, hold, possess, and enjoy any goods and chattels whatsoever." We hope that those friends to the medical profession who have property to bequeath, and upon whose generosity there are no stronger claims, will bear in mind the privilege so graciously bestowed by the King.

Nothing can be further from our intention than to enter into any lengthened enquiry of the laws or internal management of the Society. We believe that the former are, upon the whole, excellent; and that the latter is judiciously conducted. We must be allowed to hint, however, at the necessity of a very rigid adherence to the bye-laws of chap. xii., which refers to the publication or non-publication of papers presented to the Society. This is a point upon which dissatisfaction very easily arises; for who so likely to complain as the author of a rejected paper; who so likely to distort the slightest deviation from the laws into an act of premeditated partiality? The third section of the xvth chapter of the Bye-laws is thus worded: "The business of the Society, at their ordinary meetings, shall be to converse upon professional subjects, and to hear and read letters, reports, and other papers, on medicine or any of its branches." Now, in general, the meetings of the Society are wholly occupied by the reading of papers, most of which are subsequently published in the Transactions. It is true that any member may introduce a miscellaneous subject for consideration, but it has always appeared to us, and we know that others think the same, that such conversations are but coldly admitted by the president, as if they were interruptions to what we consider the comparatively dull employment of hearing papers read by the secretaries. We have heard, for example, the president cut short an interesting conversation, that the reading of a paper might be begun. More frequent discussions among the members would certainly give animation to the meetings, and render them much more attractive than they are at present; while nothing could be easier than to prevent that tone of flippant disputation which reigns in some societies, and which would certainly derogate from the character of the Royal Medico-Chirurgical.

The first paper in the volume is entitled "*Cases of Fracture of the Neck of the Femur, with the Appearances observed after Death,*" by Mr. Howship; a gentleman who has largely contributed to the pathological and practical improvement of surgery. Nine cases of fractured cervix femoris are related, not for the purpose of demonstrating the complete ossific union which occasionally takes place, but to shew the different attempts made by nature to repair the accidental injury in different cases. These cases also convey a useful lesson to the surgical practitioner as regards his diagnosis; for in each there was some variety, both in the appearance of the limb and the symptoms produced. We confine ourselves to the important facts.

Case i. *Immediate Shortening, with Eversion of the Limb.—Appearances at three weeks.* The patient had fallen on the trochanter of the right femur, and from that moment was unable to stand or move. The day after the accident, the right limb was an inch and a half shorter than the left, with eversion of the foot. She sunk, and died three weeks after the fall, without much suffering.

*Dissection.* Fracture oblique. Above, it passed through the cartilaginous margin of the head of the bone, extended obliquely round, and separated the lower part of the neck three-fourths of an inch below its junction with the head. The neck of the femur had lost half an inch of its length, by absorption. No appearance of any attempt towards union. Capsule thickened and ecchymosed. The round ligament of a livid red colour, and wasted so as to be scarcely perceptible.

Case ii. *Slight Eversion. No Shortening till six weeks after-*

*wards.—Appearances at two months,* when Mr. Howship examined the body. At first, both limbs measured the same length, the only peculiarity being a slight eversion of the leg and foot. The limb admitted of being moved and raised with facility, and without pain or crepitus. Patient suffered little; but latterly the limb imperceptibly shortened about an inch.

*Dissection.* Fracture had passed round the cervix near the head of the femur. The ligamentous expansion covering the neck of the bone was compact in texture, and much thickened. The other changes that were detected were the apparent result of inflammation. "In the abdomen, the common trunk and internal iliac veins on the left side were filled with a compact coagulum of blood: the common iliac especially was so perfectly filled, that it is not easy to understand how, towards the close of life, the blood was returned to the heart; although there had been no œdema of the lower limbs." We have in our collection an interesting specimen of a similar kind, taken from a woman who caught cold while she was menstruating. Symptoms of phlebitis came on, and she died in a short time. The abdominal cava, the iliac and femoral veins, are seen completely obstructed by firm, long coagula. In this case, also, there had been no œdematous appearance of the limbs during life.*

Case iii. *Immediate Shortening, with slight Eversion of the Limb.—Appearances at five months.* The day after the accident, great pain was felt on moving the limb, but no crepitus was discoverable. Right limb an inch shorter than the left. The patient, æt. 78, died from sloughing of the integuments covering the sacrum.

*Dissection.* Fractured head of the femur separated within the capsule, which was thickened, compact, and ecchymosed. Round ligament wasted and pulpy. The remains of the fractured neck were undergoing absorption.

Case iv. *Immediate Shortening and Eversion of the Limb.—Appearances at ten months.* A woman, æt. 79, fell on her right side. The same evening, Mr. H. found the neck of the right femur fractured, with great pain in the joint, inability to bear motion; shortening and eversion of the limb. This patient was imbecile, and would not submit to any treatment: she sank, and died ten months after the accident.

*Dissection.* Capsule of the hip thickened. The fracture had separated the neck irregularly, about half an inch below its junction with the head. Neck of the femur nearly absorbed; the part remaining being modelled into a little crutch, exactly fitted to receive and support the head, and almost entirely covered with cartilage. At the upper margin of the neck, and beneath the ligamentous investment, was a small flattened piece of cancellated bone, covered with cartilage, attached to the ligament, yet entering into the moving fabric of the new joint.

* Andral makes some interesting observations on this subject, in his Anatomie Pathologique, t. ii.—Rev.

Case v. *No immediate Shortening; slight Eversion of the Limb.—Appearances at twenty-two months.* E. B., æt. 79, fell upon the right trochanter. The same day, both limbs were of the same length; knee and foot slightly everted; hip-joint admitting free motion with pain. She could neither stand nor move the limb, complaining of pain in the joint and groin. In this instance it was at first doubtful whether fracture existed. Counter-irritation, by blisters, relieved the pain, and gave an improved power of moving and bearing on the limb: but the principal purpose for which this plan of treatment was adopted was not obtained, for gradual shortening of the limb took place. Her general health gave way, and she died.

*Dissection.* Right leg and foot much wasted, and three inches shorter than the left. Head of the femur separated from the shaft, by a fracture passing round near the margin of the head. Neck of the femur gone. From the upper part of the basis of the neck of the femur many rounded projections of bone, covered with cartilage, were put forth, over which the thickened capsule moved with facility. Round ligament wasted and shrunk.

Case vi. *Immediate Shortening, and Eversion of the Limb.* In this case, eight years after the accident, a partial, compact, ligamentous union was found on dissection. About six years after the injury, Mr. H. met the patient, a woman, æt. 70, walking without crutches. Her right hip, upon which she had fallen, was now nearly as strong as ever. She felt no pain in leaning, or even striking it with her hand.

Case vii. *Slight Eversion; no immediate Shortening.—Appearances at fourteen years.* In three months after the accident this patient, æt. 62, walked well without a stick. The injured limb was gradually shortened about two inches. On dissection, it was found that the head and neck of the bone were steadied by fibrinous bands, which gave freedom and facility of motion to the newly formed joint.

Case viii. *Immediate Shortening and Eversion of the Limb. Compact ligamentous union at five months.* M. C., æt. 66, fell on the right hip, and fractured the neck of the femur. Limb was immediately shortened two inches and a half; foot everted. Here was found, after death, a perfect example of compact ligamentous union.

Case ix. *Immediate Shortening. At first slight inversion, and subsequently eversion of the limb.* This patient partially recovered the power of moving about.

"*Cases of Warty Tumours in Cicatrices.*" By Cæsar Hawkins, Esq.

This paper commences with some very judicious comments on the various meanings assigned by different practitioners to the term *malignant,* as applied to tumours and other diseases. Hence, of course, great obscurity in pathological anatomy, which might have been avoided by more precise definition. By a malignant disease is meant, by one person, a local and fatal malady, depending upon a constitutional taint, in the way that cancer and fungus hæmatodes are invariably fatal. Another calls a disease malignant because it is incurable, and without any definite reference in his mind to the state of the constitution. Thus, lupus and the corroding ulcer of the uterus are called malignant, though the disease does not contaminate either the surrounding parts or the absorbent glands, by the formation in them of a *new structure,* like that developed in the seat of the primary disease; nor is a similar disease established in another part of the body by means of this contamination.

"Even in those diseases which are manifestly malignant, in the more confined sense in which cancer is malignant, there is great difference in the *degree* of malignancy, which the surgeon ought well to understand. Cancer of the breast very often returns in the same part, when removed by the knife; almost always affects the absorbent glands, and the appearance of a similar disease in some internal organ is always apprehended. In cancer of the scrotum, on the other hand, the removal of the diseased part is undertaken with well-grounded confidence that the disease will not reappear in the same place; the absorbent glands are often not affected, and scarcely ever is any similar disease found in the liver or any other internal organ.

"But it seems to me that we want some word for those diseases which *do form a new structure*, capable apparently of contaminating the surrounding parts, so that the removal of the whole of the altered structure is necessary, but which *do not,* as far as I know, produce any contaminating influence upon the absorbent glands, and have no tendency whatever to reappear in a distant and unconnected part of the body. Such a disease is familiar to most surgeons in the skin of the face of elderly persons, and is often, but I think erroneously, called *cancerous* and *malignant;* since, if the new structure at its basis be completely taken away, there need be no apprehension of any return of the disease, either in the same part or elsewhere: or, at least, if the new structure really possesses the nature of *cancer,* it must be clearly understood that the disease is *cancerous* and *malignant* in the very lowest degree. Of this kind also is the disease which I purpose to describe by the recital of a few cases which have fallen under my observation, and which, as far as I know, is not described in any surgical writings.

"The tumour, which I will call the *warty tumour of cicatrices*, makes its appearance in some old scar, many years after the injury which has produced it has been healed, whether a burn, a cut, or a laceration of the skin; and it arises equally from a flogging or a scald, in which the skin alone has been injured, or from a cut or gun-shot wound, which injures also the tendons or bones below the skin, and makes a more complicated cicatrix. There appears in the first place a little wart, or warty tumour, in the cicatrix, which is dry and covered with a thin cuticle, but which soon becomes moist and partially ulcerated, like the warts of mucous membranes, from which a thin, and offensive, and semi-purulent fluid is secreted. In this stage it gives no pain nor inconvenience." (P. 20.)

This first stage is shown in a preparation of a tumour removed in 1826, by Sir B. Brodie, in St. George's Hospital, from a soldier who had been repeatedly flogged. The last punishment, of one thousand lashes, had been inflicted eleven years before his admission. In the cicatrix several warts sprung up, which coalesced to form a tumour. Around this tumour the skin was of a dark livid colour, and studded with several smaller warts. The man easily recovered, and had no return of the disease.

In the second stage of the disease described by the intelligent writer, the growth of the tumour becomes more rapid, the warty appearance being in some measure lost; a more solid substance projecting from the diseased skin, which resembles fungus hæmatodes. The tumour is very vascular, and bleeds when touched.—Case 2d, also of a soldier, from military punishment, affords an example of this second stage. Case 3d, of a woman, from a scald, still further illustrates the same points. Case 4 is one of warty ulcer arising in a cicatrix upon the heel, after a wound through the tendo Achillis. Cases 5, 6, and 7, describe the same diseases from other injuries.

Mr. Hawkins concludes his brief but interesting paper by expressing a hope that the remarks he has made may enable any surgeon to recognize the disease in its early stage, when it may be removed by the knife, without losing time in using remedies, which appear to have no influence over its growth. The excision of the warty ulcer may thus prevent amputation, as in some of the cases related, or prevent the patient from being worn out by a disease that might have been eradicated while its size still allowed of the operation. Again, too, it must be important for the surgeon to know that this disease is of local origin; that it does not contaminate the constitution, so that no mischief is to be feared after its removal.

Mr. Howship next records "*a Case of Abdominal Tumour; with the Appearances on Dissection.*" In this short communication we do not perceive any points of practical interest that we could convey by the brief abstract to which we are necessarily confined.

Dr. R. Lee relates a case of "*Pulmonary Phlebitis,*" as a consequence of uterine phlebitis occurring after labour; "the only example of the disease that has yet fallen under his notice." The case is doubtless of value from its rarity.

The next contribution to the volume, "*Observations on Ulceration of the Cartilages of the Joints, and on Anchylosis,*" is from the pen of Mr. Herbert Mayo. In a paper by Mr. Key, on the Ulcerative Process in Joints, published in the eighteenth volume of the Medico-Chirurgical Transactions, two opinions are maintained: 1st, that ulceration of cartilage is more frequently dependent upon inflammation of the synovial membrane than is commonly supposed; 2d, that the inflamed synovial membrane is the agent by which cartilage is absorbed, or that cartilage is not so organized as to be capable of self-absorption, as the vascular tissues are, but, when appearing to ulcerate, is acted on and taken up either by the capsular synovial membrane, or by a false membrane superadded to the latter, with one or other of which the cartilage is in contact. The first opinion is supported by Mr. Mayo's experience; the second is opposed to the authority of William Hunter and Sir B. Brodie, and is at variance with the facts stated in Mr. Mayo's paper. Mr. Mayo's principal object, however, is not to multiply proofs of vital forces being inherent in cartilages, but to establish certain pathological differences, which he believes to have been overlooked by others, in ulcerative diseases of joints. With this purpose several cases and dissections are described which have fallen under his observation. They tend to show that articular cartilages are liable to three distinct forms of ulceration, which may be occasionally combined, but are oftener met with separately. The cases first described are instances of rapid absorption of cartilage, beginning in its synovial aspect; the new surface, if of cartilage, being smooth and unaltered in structure; if of bone, healthy; the absorption of cartilage being attended with inflammation of the capsular synovial membrane. The second series exemplifies chronic ulceration of cartilage, beginning on its synovial aspect, producing an irregularly excavated surface, with fibrous or brush-like projections of the cartilage and synovial membrane, attended with inflammation of the capsular synovial membrane, and sometimes of the same membrane where it is reflected over the cartilage; the bone, and the surface of cartilage towards it, being healthy. The third set of cases are instances of ulceration of cartilage beginning on the surface towards the bone, attended with inflammation of the adjacent surface of the bone, with inflammation of the synovial membrane, and in some instances with sensible vascularity of the cartilage itself. Mr. Mayo's paper is illustrated by three exceedingly beautiful lithographic drawings. The preparations referred to in the paper, from which the drawings were taken, may be seen in the anatomical museum of King's College.

Mr. W. H. Partridge's case of "*Singular Laceration of the Peritoneal Coat of the Uterus,*" as detected on examination after twin-labour, is worthy perusal. The "postscript" to the paper somewhat diminishes the *singularity* of the case previously related; for the author "has met" (in Velpeau's Accouchemens, probably, t. ii., p. 193 et seq., where the very same cases are referred to,) with accounts of two other cases of rupture of the peritoneal coat of the uterus, followed by death.

" *On the Chemical Constitution of Calcareous Tumours of the Uterus, and other Parts.*" By John Bostock, m.d. f.r.s. &c.

From analysis made by Dr. Bostock himself, and others of equal celebrity, the conclusion arises that these earthy concretions, in whatever part of the body they may be deposited, are nearly similar in the nature of their constituents, although with some variation in their proportions; that, in almost all instances, the phosphate of lime is the predominating substance, while, in certain cases, the carbonate would appear to be the principal ingredient.

"*Observations on Fibro-calcareous Tumours and Polypi of the Uterus.*" By Dr. R. LEE.

Dr. Lee gives a good abstract of his subject from various authorities, and adds somewhat from his own resources. Bayle has described the fibrous tumour of the uterus as fleshy at its commencement, and of a red colour, like muscular fibre, then as becoming cartilaginous, and, in the last stage, osseous. This may be the case with a few examples of the disease, Dr. Lee confesses, but he thinks not generally; and that the greater number of these tumours never exhibit a muscular or fleshy appearance at any period of their existence, but have a fibrous structure, equally distinct when not larger than a pea, and when exceeding in magnitude the head of the human adult.

"Fibrous tumours are developed either in the cellular membrane under the peritoneal coat of the uterus, or between the layers of its muscular or middle coat, or immediately between its middle and mucous coats. When situated between the peritoneum and muscular coat, they give rise to no irritation, hemorrhage, or derangement, either in the uterine functions or general health, and their existence even can only be guessed at during life. But, when they attain a large size, and occupy a great part of the abdominal cavity, they produce all the injurious consequences of enlarged ovaria, from which indeed, during life, they are distinguished with difficulty, and death usually takes place from interrupted circulation and long-continued pressure on the bladder and other contiguous viscera. Retroversion of the uterus and retention of urine have taken place in the latter stages of the disease." (P. 107.)

"There are no symptoms by which we can positively determine, during life, the presence of fibrous tumours situated between the muscular strata of the uterus: they may, however, be suspected to exist in those individuals who, being advanced beyond the middle period of life, suffer habitually from leucorrhœal discharge, who menstruate profusely, and have frequent attacks of menorrhagia, with sense of weight and irritation in the region of the uterus and adjacent organs. No alteration of structure can be discovered in the cervical portion of the uterus; but, when an examination is made, the uterus is felt larger and heavier than natural. The os uteri is neither irregular, indurated, nor painful on pressure, as it is found to be when affected with malignant disease." (P. 112.)

Dupuytren believed that uterine polypi, if abandoned to themselves, ultimately became disorganized by cancer. Dr. Lee doubts the correctness of this opinion, from his own observations; and our own experience quite confirms his scepticism as to its accuracy.

"With respect to the treatment of the various tumours which have now been described, I have a few observations to offer. Iodine, mercury, and all other remedies, have little effect either in arresting their growth or promoting their absorption. Women who have fibrous tumours formed in the walls of the uterus should avoid mechanical pressure of the hypogastrium, violent bodily exertion, and every other cause which may excite inflammation or a determination of blood to the organs within the pelvis. Where congestion has taken place, it should be removed by local bloodletting, mild cathartics, and anodynes. Profuse uterine hemorrhage should be controlled by rest in the recumbent posture, cold applications to the hypogastrium, and the internal use of the acetate of lead.

"When any of these tumours pass through the os uteri into the vagina, they may be removed by the ligature or by the knife. If the root is soft and slender, the tumour may easily be twisted off by the forceps. In the course of the last twenty years, Dupuytren states that he has removed two hundred uterine polypi by excision. Hemorrhage has only occurred twice in all the cases, and in both instances it was permanently arrested by the tampon. In eight or ten cases, after the application of the ligature, death took place from the absorption of pus into the system.

"Where the root of the tumour is large and vascular, I am of opinion that a ligature should previously be passed around it, at as great a distance from the os uteri as is compatible with the removal of the disease." (P. 132.)

"*Further Remarks on the Ulcerative Process.*" By C. Aston Key, Surgeon to Guy's.

We have, in our notice of Mr. Mayo's paper, adverted to the difference of opinion that exists between Mr. Key and that gentleman with respect to the absorption of cartilage. The doctrines which Mr. Key advances in these "Further Remarks" have not made converts of us. He conceives that cartilage has not the power of self-absorption, but that it disappears either by softening or through the action of another surface upon it. The opinions of Dr. Hunter, Sir B. Brodie, and Mr. Mayo are opposed to Mr. Key's; and, although the point is not easy of direct proof, yet we consider the case given in Mr. Mayo's paper in the same volume as decisive. This case is the subject of the first figure in the very beautiful plate attached to Mr. Mayo's paper. It appears by the narrative that in less than three weeks the cartilage had almost entirely disappeared from the astragalus in this instance; that what remained was perfectly healthy, and not softened, and perfectly adherent; and that there was no fluid in the joint, nor false membrane stretched over the articular surfaces: there was nothing, in short, in the joint *to begin the absorption of the cartilage but the cartilage itself.* We will not advert to cases in which the evidence is more ambiguous, but of which we have seen many that we have thought could bear no other construction than that which Mr. Key rejects: but, believing that it is now established that cartilage is capable of self-absorption, we venture to assert that it is more reasonable to attribute appearances to this principle, which can be explained by it, than to assume another, which is still more difficult of proof, and not more consistent with analogy.

Mr. Cock's "*Remarks on Malformation of the Internal Ear,*" and Mr. Thurnan's "*Examination of the Organs of Hearing from the Body of a Boy, æt.* 13, *born Deaf,*" require the diagrams by which they are illustrated, to render them intelligible. They are both interesting contributions to a subject of which, confessedly, we have yet much to learn. Mr. Cock remarks, that congenital deafness has been almost universally ascribed to paralysis of the auditory nerves, although there is, he believes, scarcely a case upon record in which the nerve has been found altered in its size or texture, unless through the agency of tubercles, hydatids, or some other cause producing mechanical pressure or lesion of its substance. He has examined, within the last two years, the temporal bones of five children who died in the Deaf and Dumb Asylum; and in two of these he detected such palpable deviations from the normal structure as would indicate that a congenital malformation exists oftener than is generally supposed, and therefore that to this cause many cases of deafness may reasonably be ascribed. We do not pretend to much personal knowledge of this subject; but we must observe, that no malformation of the ear is found upon examination of the majority of persons born deaf, and that it would be safer to attribute the deafness to any other cause than to that which would lead practitioners into utter despair of rendering any service, by any means.

Dr. Seymour relates three cases of *Mental Derangement* successfully treated by the acetate of morphia. The opinion that mental diseases are generally dependent upon inflammation of the brain, and that free bleeding is the proper treatment to be had recourse to, is now abandoned, except by a few routine practitioners, who never move from that degree of professional knowledge at which they quitted their lecture room. Dr. Seymour's views and doctrines are well known, and have been too frequently insisted upon by various authorities to require any additional confirmation.

"*Cases and Observations illustrative of Diagnosis when Adhesions have taken place in the Peritoneum; with Remarks upon some other Morbid Changes of that Membrane.*" By Richard Bright, M.D. F.R.S. &c.

Dr. Bright has observed on several occasions, when the circumstances of the disease had rendered it probable that adhesions might take place between the viscera and the peritoneum of the abdomen, that a very peculiar sensation has been communicated to the touch, varying between the crepitation produced by emphysema and the sensation derived from bending new leather in the hand: and, in each of the cases detailed, he found, after death, that such adhesions had existed in the parts where this sensation was discovered; but in no case has he observed the phenomenon and ascertained that the particular morbid condition did not exist; so that he infers the probability that the same adhesive process had taken place in those cases where no opportunity of examination after death was afforded. As far as we know, Dr. Bright is original in this observation; and we quite agree with him "that it may be of importance to ascertain the existence of such unnatural adhesions, as guiding and modifying not only our prognosis but our practice. At all events, let the actual utility of such information be what it may, according to the present state of our knowledge, whatever symptoms facilitate our obtaining an exact insight into the progress of disease must be satisfactory to the physician, and may hereafter prove beneficial beyond any thing we can at present foresee." Dr. Bright relates seven cases, in most of which, during the progress of abdominal disease, the "crepitus" he mentions was felt during life. The diagnosis that adhesions had taken place was verified by dissection. Mr. Copland Hutchinson relates a case, at the end of Dr. B.'s paper, in confirmation of the existence of the diagnostic symptom adverted to, when adhesions have taken place in the peritoneum.

"*On the Medicinal Properties of Creosote.*" By J. ELLIOTSON, M.D. &c.

In the early part of 1834, M. Reichenbach discovered a new principle in pyroligneous acid and all the tars, which he called Creosote, from its property of preserving animal matter: he regarded it as a cure for various diseases. Dr. E. commenced a trial with it at St. Thomas's Hospital, in July, 1834, in cases of phthisis and epilepsy. The medicine proved stimulating. If the first dose exceeded two or three drops, nausea, vomiting, vertigo, headach, and heat of the head, generally followed; but, if the dose was at first only one or two drops, many patients bore a gradual increase of it to six, ten, or twenty, without unpleasant effect. Some patients will not bear more than a fraction of a drop of creosote, while Dr. E. knew one lady who steadily augmented her dose to forty drops before it disagreed with her; but the addition of a single drop beyond this produced extreme giddiness, insensibility, and vomiting, followed by headach for several days. It appears more likely to disagree if not well diluted, though the longer it is given the less dilution frequently is necessary. At first every drop usually requires about half an ounce of water, and few persons can take many drops in much less than half a pint, without experiencing at least considerable heat in the tongue especially, and in the pharynx, œsophagus, and stomach. Dr. E.'s trials with it internally in phthisis "were perfectly unsuccessful." It is no remedy for tubercles; but, where only a single ulcer exists, or but a small number are in the lungs, and there is no disposition to further tubercular formation, it is very useful.

"One young gentleman, with a large solitary cavity in his left lung, has completely recovered, and not the slightest morbid condition is discoverable by the ear. In bronchorrhœa, or that state of the bronchial mucous membrane which consists in a profuse secretion without inflammation, I have seen its inhalation of essential service. In one instance of this affection, in which the expectoration was extremely offensive, the cure was very rapid. In asthma, also, dependent upon morbid excitability of the bronchial membrane, its inhalation is often useful. Even where it agrees perfectly well, the inhalation frequently induces a heat of the tip of the tongue." (P. 221.)

A few epileptic patients for a time had milder fits, and at longer intervals, from this remedy; but, except in one or two instances, the disease returned with its former severity, and sometimes appeared to be aggravated. The tranquillizing effect of creosote in some instances of epilepsy encouraged Dr. E. to try it in neuralgia, hysteria, and extreme nervousness; and in this class of cases it frequently succeeded. In rheumatic neuralgia, not inflammatory, Dr. E. imagines the remedy is most successful. The morbid excitability of nervous persons has been frequently abated by it in a remarkable manner; but in this description of persons, we are advised to begin with no more than half a drop, as occasionally more at first produces excitement of the head. Palpitation depending upon mere morbid excitability of the heart has yielded to it far more than other remedies. The "extraordinary power" which the creosote possesses, of arresting vomiting, when not dependent on inflammation or structural disease, Dr. E. "considers to be perfectly established." During inflammation, the stimulant power of the medicine does harm. If structural disease exists, it aggravates pain, even if it arrest the vomiting. A few persons are so disgusted with the smell of tar, that any attempt to take the creosote makes them sick; but, with these exceptions, Dr. E. "knows no other medicine at all to be compared with it in arresting vomiting." He has often seen it succeed after the failure of prussic acid, the most powerful remedy he was previously acquainted with in such cases. In different cases of vomiting different doses are required. In colic and enteritis, it arrests the vomiting long before the bowels are opened, and purgatives are thus retained which were before rejected. Dr. E. has tried it in only one case of vomiting from pregnancy, and with success. In sea-sickness also it has been found advantageous, but at present upon a small scale. Its power, we are assured, is equally great over nausea. When properly given in cases of nausea or vomiting, without inflammation or structural disease of the stomach, Dr. E. has only known it to fail in one remarkable case, which he relates. Having ascertained this power, experiments were made to determine, whether, like prussic acid, creosote would prevent other medicines from exciting nausea and vomiting. The result is thus confidently expressed:

"I find it, by daily experience, even to surpass prussic acid in this particular. I have enabled the stomach to bear hydriodate of potass, sulphate of copper, sulphate of iron, and many diuretics, &c., in much larger quantities than those previously rejected. Just as I have often seen it arrest vomiting where prussic acid had failed, so I have seen it enable the stomach to bear medicines when they had been rejected in spite of prussic acid." (P. 230.)

No action is produced upon the bowels by the creosote. It sometimes augments the quantity of urine. Dr. E. once saw it, in doses of a minim three times a day, cause micturition nine times in an hour. In another case, in doses of three minims, it produced severe strangury. In three cases of diabetes, it was thought to do good. Dr. E. speaks favorably, too, of its external application in ulcers that require a stimulus, and in sloughs with offensive discharge. In pruritus podicis, in toothach applied pure, and in porrigo, it has sometimes been found serviceable. Dr. E. adds, in a postscript, that he has witnessed "its extraordinary power over nausea and vomiting in at least fifty more cases, in which the stomach was neither inflamed nor diseased." In two cases of glanders contracted from a glandered horse, "the sedulous injection of a weak solution of creosote up the nostril removed the whole of the symptoms in a few weeks."

So far we have confined ourselves to an abstract of Dr. E.'s warm eulogiums respecting the creosote, but we cannot quit the subject without observing that, in other and equally judicious hands, with all the required conditions present in the cases in which it was given, it has fallen very far short of the virtues ascribed to it in his paper. We sincerely hope that further experience may confirm Dr. E.'s opinions: we fear, nay we believe, it will not; and that, even in those cases—nausea and vomiting, without inflammation or organic disease,—in which he much extols its powers, the result of accumulated evidence will be, that it sometimes succeeds, more frequently fails, and is even occasionally injurious. The creosote is at least not to be trifled with, with impunity, and we caution practitioners in general, from what we know of its effects, to prescribe it with great circumspection.

The case next related is by Dr. Robert Lee, "*on the Functions of the Fœtal Kidney.*"

Mr. Earle's "*Observations on Fractures of the Bones of the Pelvis*" well merits the attention of surgeons. Five interesting cases are related, all of which occurred in Mr. Earle's public and private practice.

The volume concludes with a paper by Dr. Sims, of much pathological and practical value, "*on Serous Effusion from the Membranes and into the Ventricles of the Brain, and its Connexion with Apoplexy and other Diseases of the Brain.*"

After having bestowed upon this volume a very attentive perusal, we rise from it with the impression that it is somewhat inferior, upon the whole, to most of the previous "Transactions." Several of the papers contain excellent matter, and are very neatly drawn up; others, upon which we could fix, contain little that is new, and have certainly no fair claim to the space they occupy.

